# Anisotropy of impact ionization in WSe_2_ field effect transistors

**DOI:** 10.1186/s40580-023-00361-x

**Published:** 2023-03-17

**Authors:** Taeho Kang, Haeju Choi, Jinshu Li, Chanwoo Kang, Euyheon Hwang, Sungjoo Lee

**Affiliations:** 1 SKKU Advanced Institute of Nanotechnology (SAINT), Sungkyunkwan University, Suwon, 16419 South Korea; 2 Department of Nano Science and Technology, Sungkyunkwan University, Suwon, 16419 South Korea; 3 Department of Nano Engineering, Sungkyunkwan University, Suwon, 16419 South Korea

**Keywords:** Impact ionization, 2D layered material, WSe_2_ field effect transistor, Carrier multiplication, Steep switching

## Abstract

Carrier multiplication via impact ionization in two-dimensional (2D) layered materials is a very promising process for manufacturing high-performance devices because the multiplication has been reported to overcome thermodynamic conversion limits. Given that 2D layered materials exhibit highly anisotropic transport properties, understanding the directionally-dependent multiplication process is necessary for device applications. In this study, the anisotropy of carrier multiplication in the 2D layered material, WSe_2_, is investigated. To study the multiplication anisotropy of WSe_2_, both lateral and vertical WSe_2_ field effect transistors (FETs) are fabricated and their electrical and transport properties are investigated. We find that the multiplication anisotropy is much bigger than the transport anisotropy, i.e., the critical electric field (E_CR_) for impact ionization of vertical WSe_2_ FETs is approximately ten times higher than that of lateral FETs. To understand the experimental results we calculate the average energy of the carriers in the proposed devices under strong electric fields by using the Monte Carlo simulation method. The calculated average energy is strongly dependent on the transport directions and we find that the critical electric field for impact ionization in vertical devices is approximately one order of magnitude larger than that of the lateral devices, consistent with experimental results. Our findings provide new strategies for the future development of low-power electric and photoelectric devices.

**Graphical Abstract:**

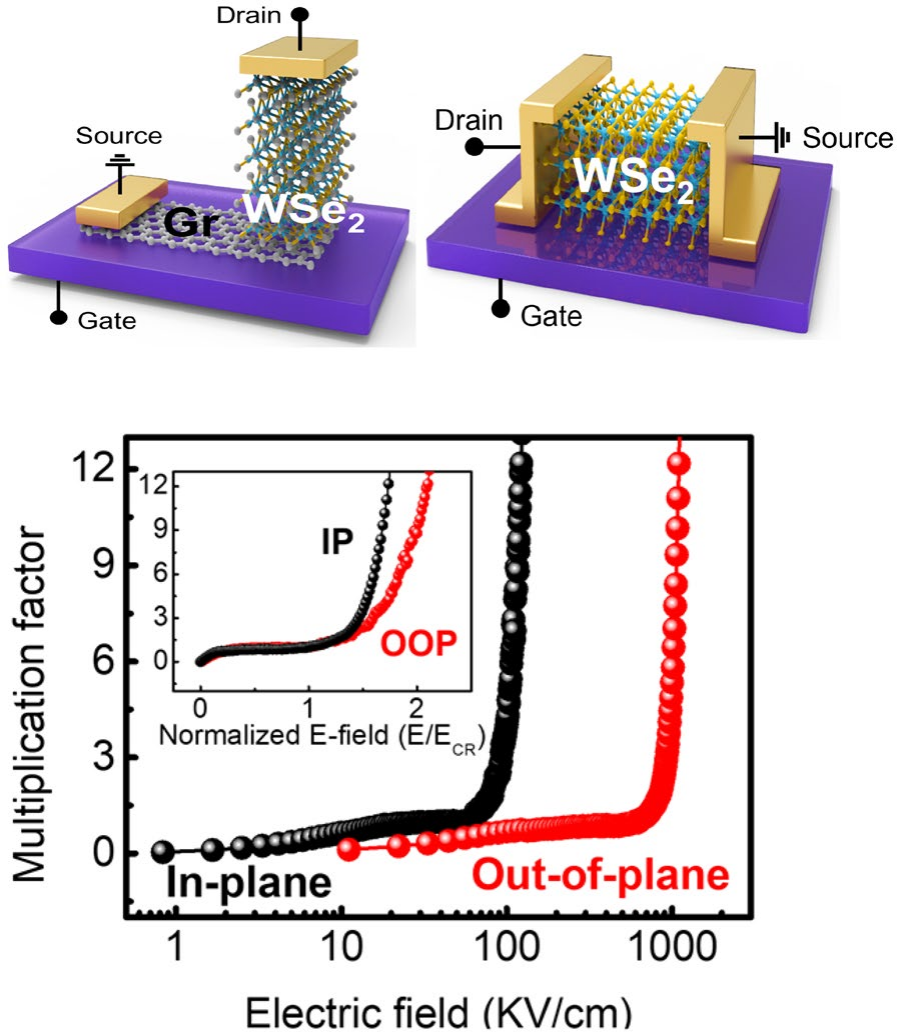

**Supplementary Information:**

The online version contains supplementary material available at 10.1186/s40580-023-00361-x.

## Introduction

For future energy-efficient, low-power devices, numerous efforts have been made to overcome the room-temperature subthreshold slope (SS) of 60 mV/dec using quantum tunneling [1–4], mechanical switching [5], and negative-capacitance [6, 7]. Additionally, impact ionization has attracted significant attention because new functional applications employing it have achieved an ultralow subthreshold swing [8] and sensitive photodetection [9, 10], with low power consumption. Impact ionization is a carrier multiplication process wherein sufficiently accelerated carriers collide with a lattice and generate more free carriers. The generated carriers repeat this process, thus creating more electron–hole pairs that are accelerated under a high electric field, consequently inducing avalanche multiplication and an abrupt current increase. The efficiency of the impact ionization process depends on the intrinsic property of materials [11]. Therefore, the discovery of novel materials with low critical electric field (E_CR_) for impact ionization is important in achieving energy-efficient electric/photoelectric devices. The impact ionization has been studied in conventional materials (such as Si, Ge, InAs, and GaAs) [12–18], but the device application has been limited due to a high driving voltage owing to the high E_CR_ of these materials.

Recently, advances in 2D layered materials and their heterostructures have re-spurred investigations into impact ionization. Their unique transport characteristics and structural features have been revealed and the layered materials are useful in future generations of nanoelectronic devices. Due to the charge confinement and low dielectric screening effect efficient carrier multiplication has been achieved in 2D layered materials [19]. Recent developments in the fields of impact ionization-based electronic [20, 21] and photonic [22, 23] devices employing 2D layered materials and their heterostructures have proven this. The transport directions underlying their operation can be classified into lateral and vertical transport, essentially originating from the fundamental anisotropy between the in-plane and out-of-plane 2D layered materials [24]. Differences in electrical and optical properties owing to their unique anisotropy have already been reported [25, 26], and these materials have the potential to exhibit different impact ionization characteristics according to the carrier transport direction. Therefore, to achieve energy-efficient carrier multiplication, further evaluation of carrier transport-induced direction-dependent impact ionization in single 2D layered materials is crucial.

In this paper we investigated the directional dependence of the impact ionization characteristics of the 2D layered material WSe_2_. To study the anisotropy of the impact ionization characteristics, both lateral and vertical WSe_2_ FETs were fabricated, and their in-plane and out-of-plane carrier transport were studied. By applying sufficiently high drain voltages, the I–V characteristics of both devices were analyzed and their critical electric fields (E_CR_) for impact ionization and multiplication factors (M) was determined. Unexpectedly, we find the multiplication anisotropy is much bigger than the transport anisotropy, i.e., the critical field of the vertical WSe_2_ FETs was an order of magnitude higher than that of the lateral FETs, and much lower multiplication factor was observed in the vertical devices. These results show a significant difference whether the carriers travel in-plane or out-of-plane. Furthermore, a theoretical analysis was performed using Monte Carlo simulations to understand the experimentally measured anisotropy of impact ionizations. By considering all possible hot carrier relaxation processes, the scattering rates and average carrier energies for both the lateral and vertical FETs were calculated. In general, impact ionization occurs when the average energy of the carriers exceeds the band gap energy (E_g_) of a material (here, the band gap energy of bulk WSe_2_ was estimated to be 1.0 eV). Based on our calculations, the electric fields required for impact ionization in out-of-plane transport were hundreds of kV/cm, whereas for in in-plane transport, the fields required were tens of kV/cm, which are consistent with our experimental results. This study provides a deeper understanding of the anisotropic impact ionization characteristics of 2D layered materials and suggests a new strategy to achieve energy-efficient carrier multiplication via appropriate impact ionization, which can contribute to the enhancement of future low-power devices.

**Fig. 1:**
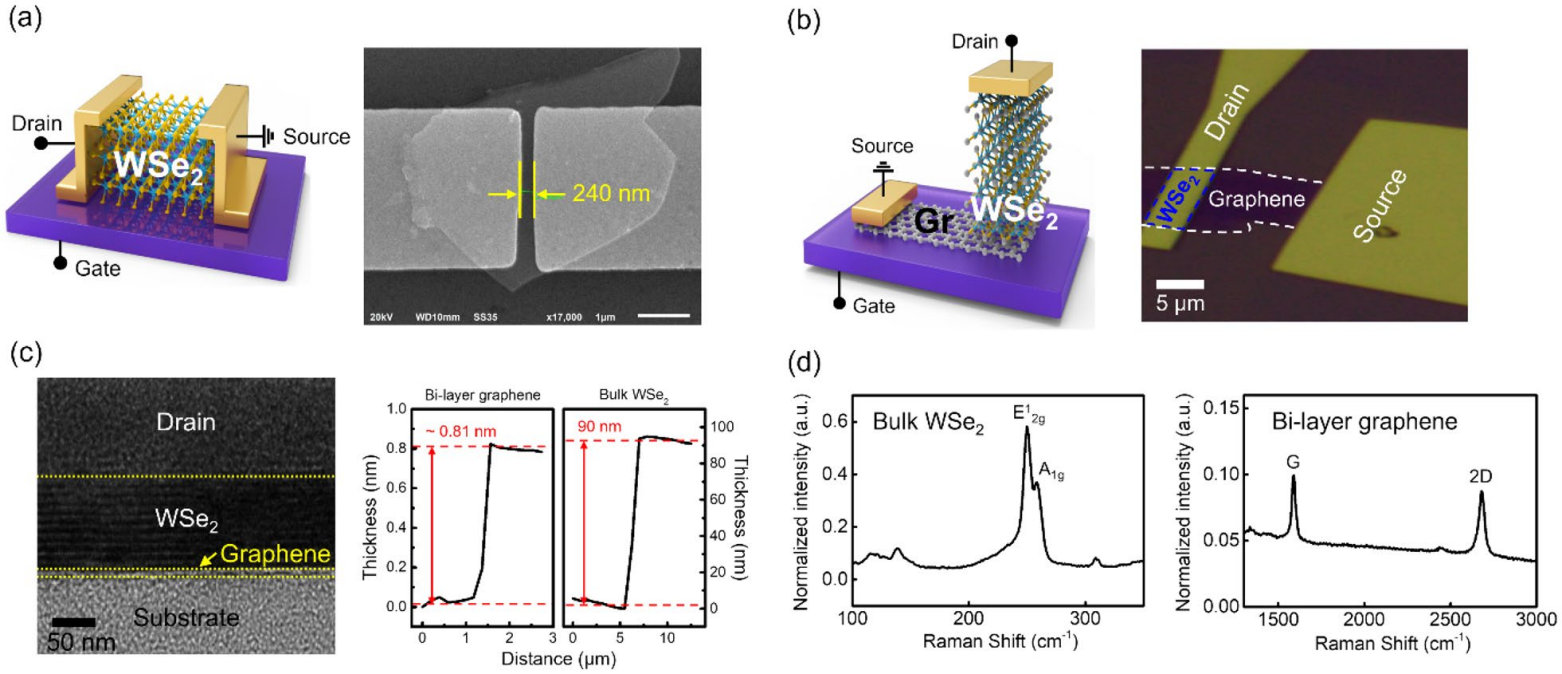
Device structures and sample characterization.** a** Left panel: schematic of the lateral WSe_2_ FET. Right panel: SEM image of the lateral WSe_2_ FET, with the channel length indicated. **b** Left panel: schematic of the vertical WSe_2_ FET employing a WSe_2_/graphene heterostructure. Right panel: OM image of the vertical WSe_2_ FET. **c** Corresponding HR-TEM image and AFM data indicating the thickness of the WSe_2_/graphene heterostructure (the thickness of the WSe_2_ used in the lateral FET is also same as 90 nm). The stacked structure consists of bi-layer graphene, bulk WSe_2_, and an Au electrode as the drain. **d** Raman spectra of mechanically exfoliated WSe_2_ and bi-layer graphene used for in the fabrication of devices

## Results and discussion

Figure 1a and b show schematics and corresponding optical images of the lateral and vertical WSe_2_ FETs, respectively. A lateral FET was fabricated by forming the source-drain via the EBL process on 90 nm thick WSe_2_, which was transferred onto the Si substrate. To fabricate the vertical WSe_2_ FET, a WSe_2_/bilayer graphene heterostructure was used. After bilayer graphene (BLG) and bulk WSe_2_ samples were prepared by mechanical exfoliation, the WSe_2_/BLG heterostructure was dry-transferred onto a 285 nm SiO_2_/p^++^–Si substrate. In the vertical WSe_2_ FET, the BLG acted as a source electrode and the gold contact on top of WSe_2_ acted as a drain electrode. Thus, the current flowed vertically between the bottom BLG electrode and the top Au electrode through the semiconducting bulk WSe_2_ channel (see Additional file 1: Section 1a for a detailed description of the fabrication process). The left panel of Fig. 1c shows a cross-sectional HR-TEM image of the stacked Au/WSe_2_/BLG structure. The Au electrode (top), WSe_2_ channel (middle), and BLG (bottom) were identified. The thickness of the samples, which was measured by atomic force microscopy (AFM), is shown in the right panel of Fig. 1c. The thickness of WSe_2_, which was the channel length in the vertical FET, was confirmed to be approximately 90 nm. The Raman peaks shown in Fig. 1d confirm that the samples were composed of uniform bulk WSe_2_ and bilayer graphene.

**Fig. 2:**
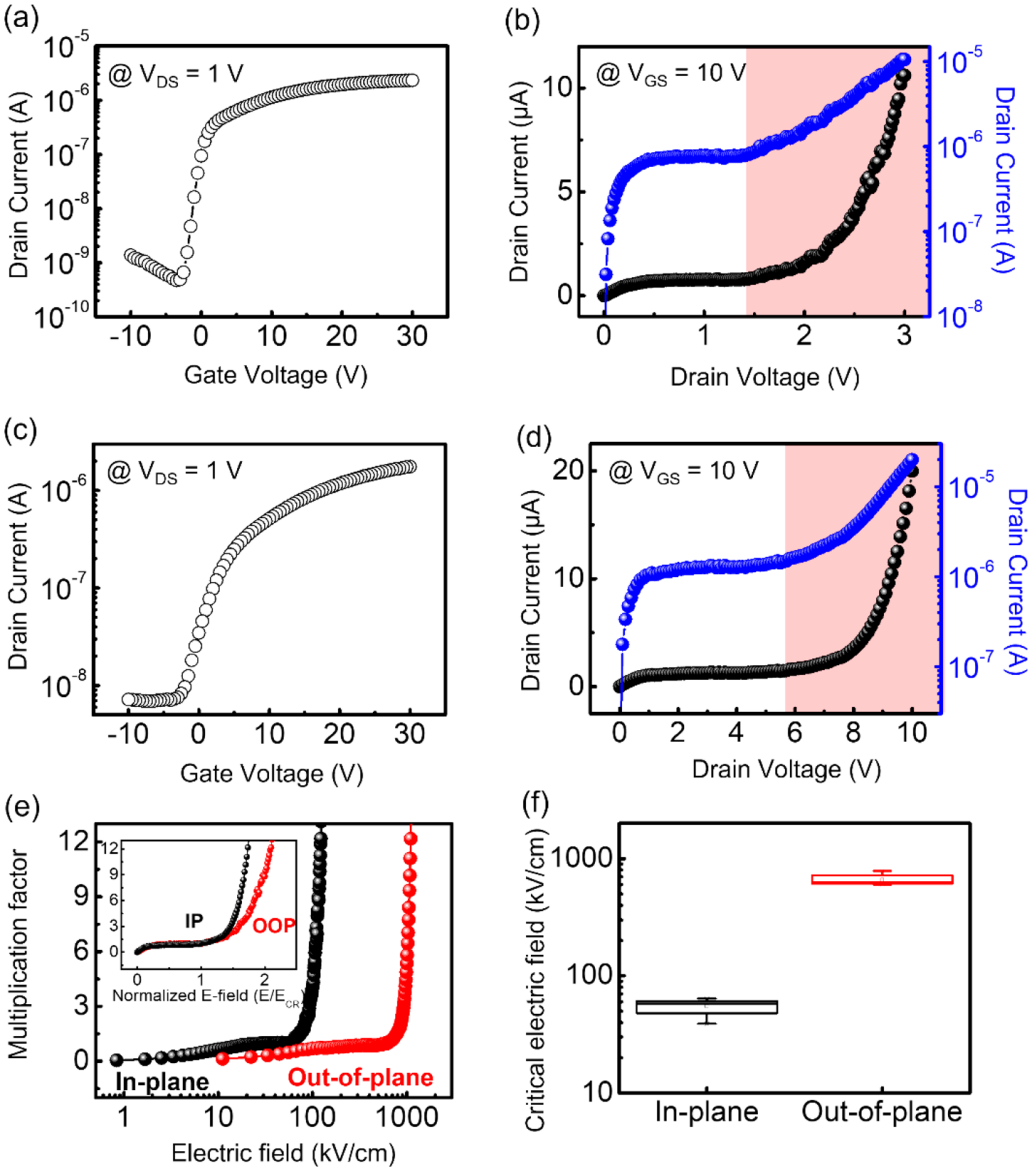
Impact ionization characteristics for different carrier transport directions.** a** Representative transfer curve of the lateral FET with a 240 nm WSe_2_ channel. **b** Output characteristics of the in-plane direction which shows impact ionization occurring above 1.4 V. **c** Representative transfer curve of the vertical FET employing 90 nm thick WSe_2_. **d** I_DS_–V_DS_ characteristics of the out-of-plane direction exhibiting impact ionization occurring above 5.7 V. **e** Calculated multiplication factors for each direction as a function of the electric field. For impact ionization to occur in the out-of-plane direction, an electric field that is approximately ten times larger than that of the in-plane is required. (Inset: multiplication factors as a function of the electric field, normalized by the E_CR_.) **f** Distributions of the critical electric fields in tens of devices

Figure 2a shows a representative transfer curve (at V_DS_ = 1 V) for the lateral WSe_2_ FET. Typical ambipolar transport in WSe_2_ was observed, that is, current is minimum at the charge-neutral point (V_CNP_ = − 4 V for this device) and as |V_GS_ − V_CNP_| increased the carrier concentration increased owing to electrostatic doping, and consequently, the drain current increased. Figure 2b shows the measured current as a function of the drain voltage at a fixed gate voltage (V_GS_ = 10 V). As shown in Additional file 1: Sections 2a and b, our devices have similar carrier concentration (~ 10^12^ cm^−2^ at V_GS_ = 10 V) values ​​in both lateral and vertical FETs. In this regard, we applied a fixed gate voltage (10 V) in both devices to compare the impact ionization properties. At low biases (V_DS_ < 0.3), the measured current increased with increasing drain voltage, but the saturation of current was observed in the range 0.3 < V_DS_ < 1.4 V. The saturation indicates a thermal equilibrium state in which the net rate of energy exchange of carriers produced by both emitting and absorbing phonons is zero [27, 28]. At higher bias voltages (i.e., V_DS_ > 1.4 V), the current increased again, and this behavior is known to be induced by the impact ionization process [29, 30]. Under strong biases (equivalently high electric fields), the carriers gained sufficient energy to generate new electron–hole pairs by impact ionization. Furthermore, reversible characteristics during multiple V_DS_ sweeps were obtained by limiting the electric field across the channel, thus preventing permanent breakdown by Joule heating (see Additional file 1: Section 3b).

Figure 2c and d show the transfer curve (drain current as a function of gate voltage at a fixed drain voltage) and the I–V characteristics of the vertical WSe_2_ FET, respectively. We note that the gate voltage in the vertical FET controls the Schottky barrier height (SBH) at the contact; thus, unipolar n-type transport characteristics were observed (see Additional file 1: Sections 2c and d). As shown in Fig. 2d, the I–V characteristics of the vertical WSe_2_ FET can be divided into three regions depending on the bias voltage. We note that the current saturation at intermediate drain voltages (0.8 < V_DS_ < 5.6 V) was unrelated to the carrier overshoot effect (see Additional file 1: Section 2e for a description of the overshoot effect). For the high-bias region (V_DS_ > 5.6 V), the channel current increased after saturation, i.e., impact ionization occurred in the out-of-plane direction.

Figure 2e shows the multiplication factor ($M={I}_{DS}/{I}_{sat}$, where ${I}_{DS}$ is the drain current and ${I}_{sat}$ is the saturation current) as a function of the electric field for both lateral and vertical WSe_2_ FETs. A significant difference in the magnitude of the critical electric field (*E*_*CR*_) at which the impact ionization process begins was observed. *E*_*CR*_ was estimated as 58 kV/cm for the lateral and 633 kV/cm for the vertical WSe_2_ FET. A higher multiplication factor was observed in the in-plane direction of impact ionization compared with the out-of-plane direction. In the inset of Fig. 2e, the scaled multiplication factor (*M*) is shown as a function of the electric field (normalized by the *E*_*CR*_ (*E/E*_*CR*_)). The figure indicates that the multiplication factor is more sensitive to the electric fields in the lateral device. The critical electric fields of several different devices were obtained; Fig. 2f shows the measured critical electric fields as well as variations in the field. The critical electric fields of the vertical FETs were an order of magnitude larger than those of lateral FETs, thus indicating that carrier generation as a result of impact ionization occurs more efficiently in the in-plane direction than in the out-of-plane direction. Several possible reasons can explain the anisotropy (i.e., directional dependence) of the carrier multiplication from impact ionization: different electrostatic doping, the effect of interlayer transport, or scattering mechanisms depending on the carrier transport direction. We investigated the total interlayer resistance (R_interlayer_) in the Au–WSe_2_-graphene vertical structure. The total interlayer resistance was calculated using the following equation: total R_interlayer_ = $\rho$_interlayer_ (d_interlayer_/A) (N_interlayer_), where $\rho$_interlayer_ is interlayer resistivity, d_interlayer_ is the interlayer distance, A is the area of the conducting system, and N_interlayer_ is the number of interlayers. Considering the $\rho$_interlayer_ = 2.0 $\varOmega$mm [31], d_interlayer_ = 0.651 nm, and N_interlayer_ = 42 layers, we calculated the value of the R_interlayer_ to be approximately $4 \varOmega$. This small value indicates that the voltage applied to the interlayer is negligible, which implies that it cannot be a significant reason for the large electric field of vertical transport. Therefore, we expect that the directional dependence of the impact ionization in WSe_2_ is originated from the different scattering mechanisms.

To investigate the origin of this difference in the impact ionization properties depending on the carrier transport direction, the hot-carrier transport was analyzed in both lateral and vertical WSe_2_ FETs using Monte Carlo simulations [32]. The impact ionization is closely related to the relaxation of hot carriers. Given that scattering by phonons is the most important relaxation process for hot carriers, all possible phonons were considered in the relaxation process. All other scatterings (i.e., impurities, vacancies, defects, etc.) contribute little to relaxation because such scatterings are elastic [27, 28]. We calculated the scattering rate by phonons for two different experimental setups and compared the results to understand the anisotropy of the impact ionization.

**Fig. 3:**
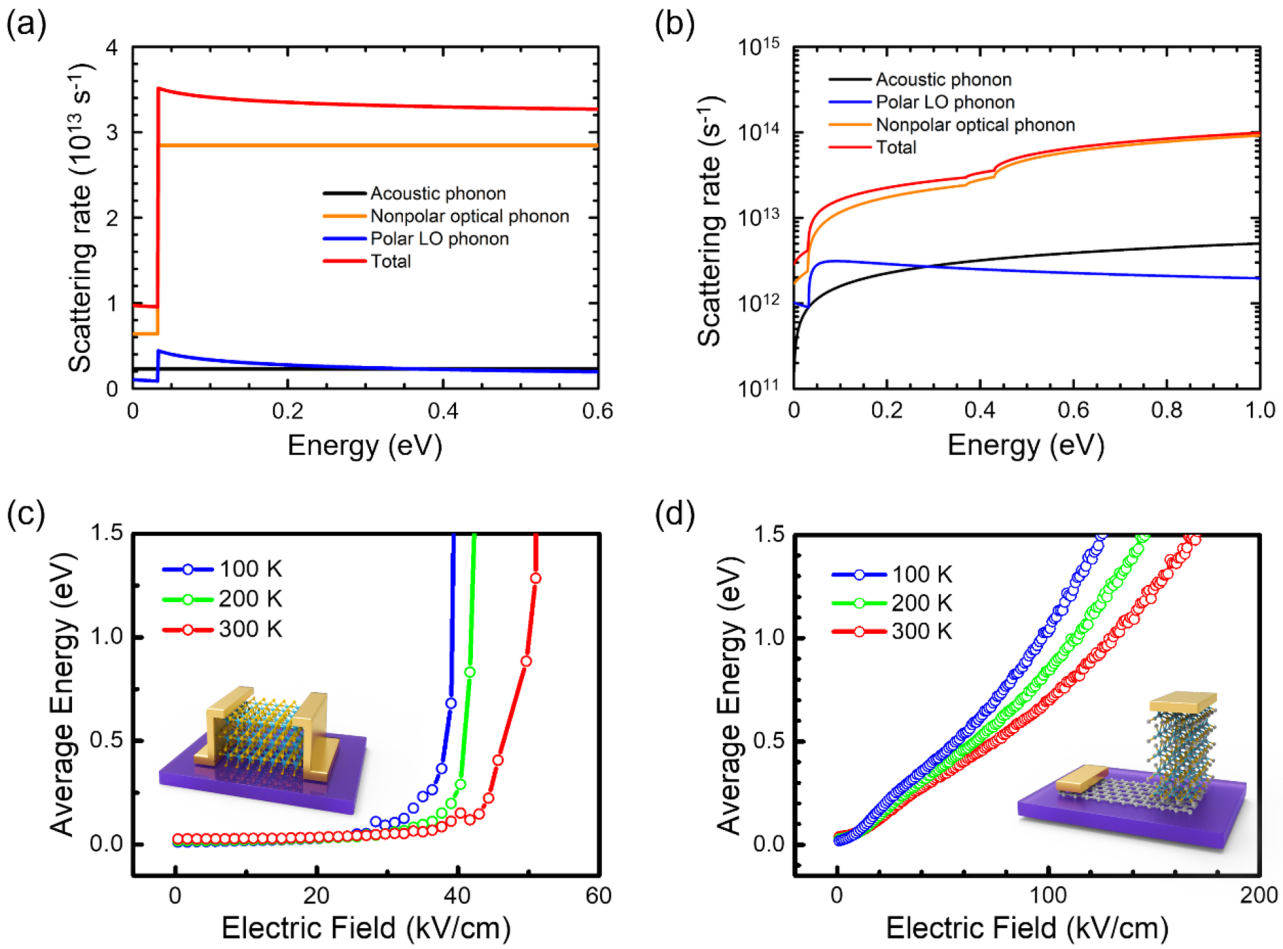
Theoretical analysis of impact ionization.** a** Two-dimensional scattering rates for different phonon scatterings as a function of the carrier energy at T = 300 K. The acoustic phonon deformation potential is 2 eV and the nonpolar optical phonon deformation potential is 4 × 10^8^ eV/cm. **b** Three-dimensional scattering rates for different phonon scatterings as a function of the carrier energy at T = 300 K in the Q conduction valley. The acoustic phonon deformation potential is 3 eV and the nonpolar optical phonon deformation potential is 5 × 10^8^ eV/cm. **c** Monte Carlo simulated average carrier energy for in-plane transport as a function of the electric field at T = 100, 200, and 300 K. **d** Monte Carlo simulated average carrier energy for out-of-plane transport as a function of the electric field at T = 100, 200, and 300 K. The electric field required to reach E = 1.5∙E_g_ = 1.5 eV, the carrier energy for impact ionization, is exceptionally large for out-of-plane transport compared with in-plane

Figure 3a and b show the energy-dependent scattering rates for the in-plane and out-of-plane transport in WSe_2_, respectively. The contributions of acoustic phonons, polar LO phonons, and nonpolar optical phonons to the total scattering rate are shown in Fig. 3. We found that the total scattering rate was dominated by nonpolar optical phonons, as shown in Fig. 3a and b. The sharp cusps at 32 meV arose from the emission of the optical phonons. The main difference between lateral and vertical transport was the degree of freedom of the scatterings. The lateral FETs were dynamically 2D under a gate voltage; that is, the motion of electrons or holes was confined in the vertical direction and they were free to move in a unique in-plane dimension [33]. In the vertical structure, carriers moved three-dimensionally with anisotropic effective masses [34]. Consequently, the determined scattering rates, owing to identical phonon scattering, yielded different results for each device configuration (see Additional file 1: Section 4a for details).

In Fig. 3c, the calculated average energy of electrons in the lateral WSe_2_ FET is plotted as a function of the electric field for different temperatures ranging from 100 to 300 K. At low electric fields, the average electron energy increased extremely slowly because of the balanced thermal equilibrium, wherein carriers both emit and absorb phonons. However, at high electric fields, the average electron energy dramatically increased, exceeding the band gap energy of the material at the critical electric field (E_CR_). Thus, at the critical electric field, impact ionization occurred; E_CR_ for the lateral FET was approximately 50 kV/cm at 300 K. As shown in Fig. 3c, no energy balance mechanism was possible at high electric fields (above the E_CR_); an energy runaway occurred, which is believed to be the cause of the impact ionization process. For comparison, the average carrier energy in the vertical transport system was calculated, and is shown in Fig. 3d as a function of the electric field at different temperatures ranging from 100 to 300 K. In contrast to the sharp cusp that appeared for the lateral devices (Fig. 3c), the calculated average electron energy of the vertical device gradually increased and reached the impact ionization threshold energy (E_I_). This behavior can be explained by the phonon scattering mechanisms that occurred in vertical devices; that is, nonpolar optical phonons gave rise to the energy-dependent scattering rate owing to the 3D density of final states (DOS). More importantly, the total scattering rate of nonpolar optical phonons monotonically increased over all energy ranges. Therefore, nonpolar phonon interactions with carriers are crucial in determining the behavior of hot electrons in the out-of-plane transport system. Owing to this energy runaway process, E_CR_ reached hundreds of kV/cm in the vertical devices. In addition, our theoretical analysis showed that the temperature plays a more important role in out-of-plane impact ionization, wherein the average electron energy significantly increased at lower temperatures.

**Fig. 4:**
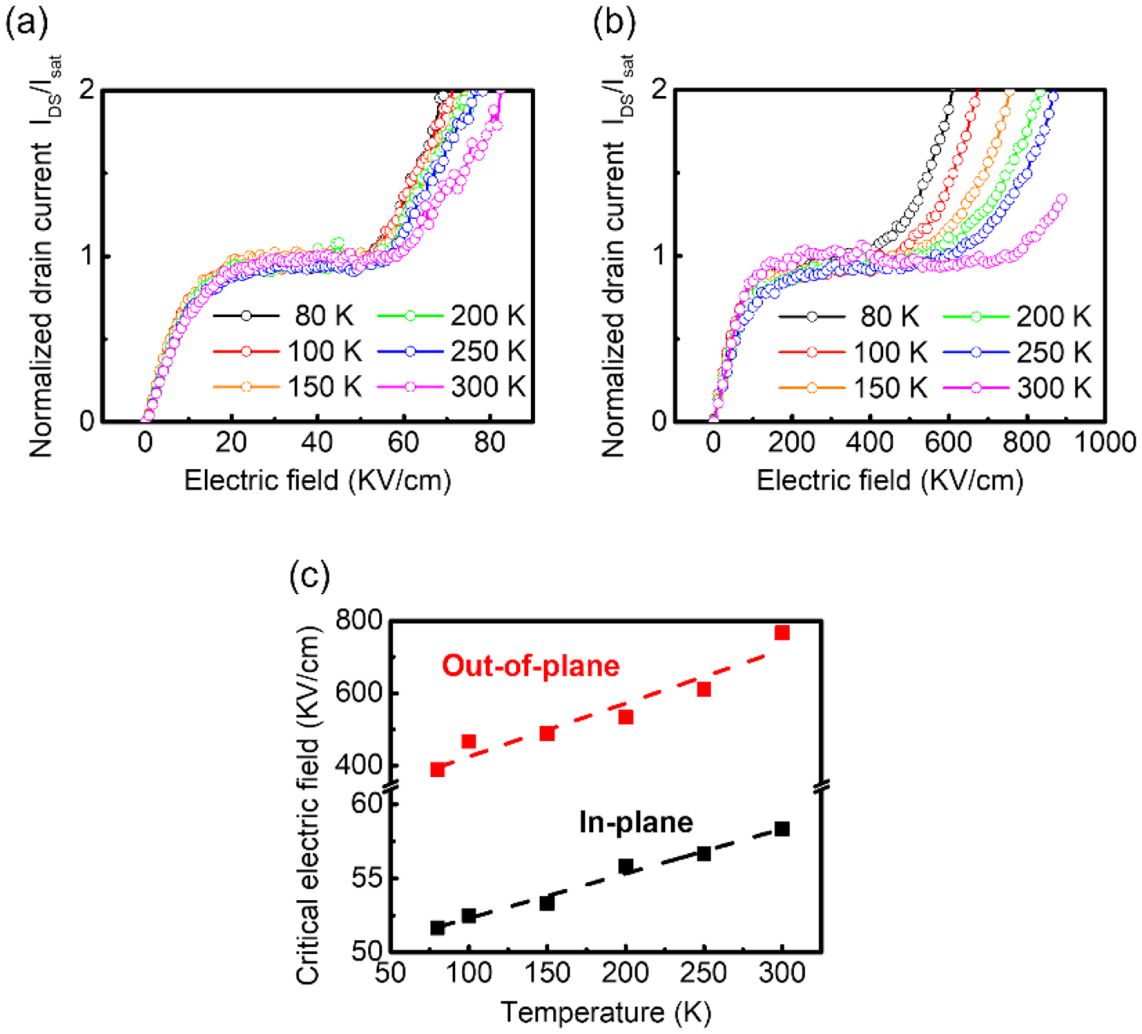
Temperature-dependent impact ionization for in-plane and out-of-plane transport. Drain current normalized by the saturation current for **a** in-plane transport and **b** out-of-plane transport at various temperature ranging from 80 to 300 K. **c** Critical electric fields of in-plane (black square) and out-of-plane (red square) transport as a function of the temperature. The critical electric field decreases with decreasing temperature; the decrease is very slight for in-plane transport. Conversely, for out-of-plane transport, the decrease in the critical electric field due to the temperature drop is relatively large because optical phonon scattering is dominant in this transport system

To investigate the temperature dependence of the critical electric field, the I_DS_–V_DS_ characteristics of both devices were measured at various temperatures ranging from 80 to 300 K. The channel currents (normalized by the saturation current) as a function of the applied electric field are shown in Fig. 4a and b for the in-plane and out-of-plane directions, respectively. For both directions, a decrease in the critical electric field was observed with decreasing temperature. However, although the change in E_CR_ was insignificant for the lateral device (approximately 10%) in the measured temperature range, the change was approximately 100% for the vertical device. Conversely, for out-of-plane impact ionization, a relatively large decrease was observed in the critical electric field with decreasing temperature. Figure 4c shows the critical electric field as a function of temperature. As shown in Fig. 4c, the critical electric fields increased linearly with temperature for both devices; however, for all temperatures, a larger electric field was required to induce impact ionization in out-of-plane transport than in in-plane transport. The observed strong temperature dependence of the critical field in out-of-plane transport is induced by the optical phonons acted as the dominant scattering mechanism in the out-of-plane transport. As the effect of optical phonon scattering is extremely strong in the out-of-plane transport for all temperatures, a large E_CR_ of 389 kV/cm was obtained even at 80 K. Based on our theoretical and experimental analyses, to initiate the impact ionization in vertical WSe_2_ devices exceptionally high electric fields of hundreds of kV/cm is required, which is ten times higher than the fields required for lateral devices.

## Conclusion

In conclusion, both the lateral and vertical WSe_2_ FETs were investigated to understand the anisotropy of the impact ionization in the layered 2D materials. The results revealed significant differences depending on whether the carrier travels in-plane or out-of-plane. Additionally, the critical electric field (E_CR_) for impact ionization in the out-of-plane direction was an order of magnitude larger than that in the in-plane direction. This difference arose from the relaxation of hot carriers via phonon scattering. Furthermore, the temperature dependence of the critical electric fields was investigated for impact ionization. Evidently, although the critical fields increased with temperature for both transport directions, the temperature dependence of the field was much stronger in out-of-plane transport. This study helps to understand carrier transport direction-dependent impact ionization in 2D layered materials and provides a new strategy to improve the carrier multiplication efficiency via suitable impact ionization, which can contribute to future low-power devices.

## Methods

### Device fabrication

WSe_2_ flakes and bilayer graphene were exfoliated from bulk crystals using the Scotch tape method and were dry-transferred onto a 285 nm SiO_2_/p^++^–Si substrate using PDMS. The exfoliation and transfer processes were performed in a controlled glove box environment to prevent any external perturbations. The thicknesses of the materials were first measured using an optical microscope and then accurately determined using AFM. For further thickness control or to define channels (to eliminate unwanted current pathways apart from the target transport direction), an inductively coupled plasma etching process was performed using Ar/SF_6_ gas. Electron-beam deposition and electron-beam lithography were used to form and pattern the Au (50 nm) source and drain electrodes in a high-vacuum chamber (5 × 10^− 7^ Torr). Please see Additional file 1: Supplementary section 1a for a detailed description of the fabrication process.

### Characterization

Optical microscopy (OM; Olympus, BX51M) and field emission scanning electron microscopy (FE-SEM; JEOL, JSM7500F) were used to observe the sizes and shapes of the prepared samples and the fabricated devices. Raman spectra were acquired at a laser excitation wavelength of 503 nm to characterize the quality of the samples. The thickness of the flakes was determined via AFM with an atomic force microscope (Park Systems Corp., NX-10) in noncontact mode with PPP-NCHR probe tips (Nanosensors). The electrical properties of the lateral and vertical WSe_2_ FETs were measured using a Keithley 4200 parameter analyzer at various temperatures by employing a hot chuck controller (MS Tech, MST1000H) and a cryostat system (MS Tech, VX7).

## Supplementary Information

**Additional file 1: Table S1.** Conduction band parameters of multilayer WSe2.

## Data Availability

The datasets used and/or analyzed during the current study are available from the corresponding author on reasonable request.
