# Supplementary material for: Anisotropy of impact ionization in WSe2 field effect transistors
Source: Nano Converg. 2023 Mar 17;10:13. doi: 10.1186/s40580-023-00361-x (PMC10023822; doi:10.1186/s40580-023-00361-x)
Supplement: Supplementary file 1 — Additional file 1: Table S1. Conduction band parameters of multilayer WSe2. [file 40580_2023_361_MOESM1_ESM.docx]

Supporting Information

Anisotropy of Impact Ionization in WSe_2_ Field Effect Transistors

Taeho Kang^1^, Haeju Choi^1^, Jinshu Li^1^,Chanwoo Kang^1^, Euyheon Hwang^1,2^, and Sungjoo Lee^1,2^*

^1^SKKU Advanced Institute of Nanotechnology (SAINT), Sungkyunkwan University, Suwon 16419, Korea

^2^Department of Nano Science and Technology, Sungkyunkwan University, Suwon 16419, Korea

^3^Department of Nano Engineering, Sungkyunkwan University, Suwon 16419, Korea

E-mail: leesj@skku.edu; euyheon@skku.edu.

Contents

**Supplementary section 1: Device fabrication**

**a. Fabrication process**

**Supplementary section 2: Bi-layer graphene as a bottom electrode**

**a. Negligible effect of bi-layer graphene layer on carrier concentration**

**b. Gate bias modulation through bi-layer graphene**

**c. Tuning the Schottky barrier at the WSe_2_/graphene interface through gate voltage**

**d. Electrical properties of bi-layer graphene**

**e. Excluding the effect of velocity overshoot**

**Supplementary section 3: Demonstration of impact ionization**

**a. Analysis of impact ionization**

**b. Reversible output characteristics during V_DS_ sweeping**

**c. Reliability of the impact ionization process**

**Supplementary section 4: Monte Carlo simulations**

**a. Scattering mechanisms in WSe_2_**

**b. Monte Carlo simulation result for out-of-plane impact ionization**

**c. Monte Carlo simulation result for in-plane impact ionization**

**Supplementary section 1. Device fabrication**

**a. Fabrication process**


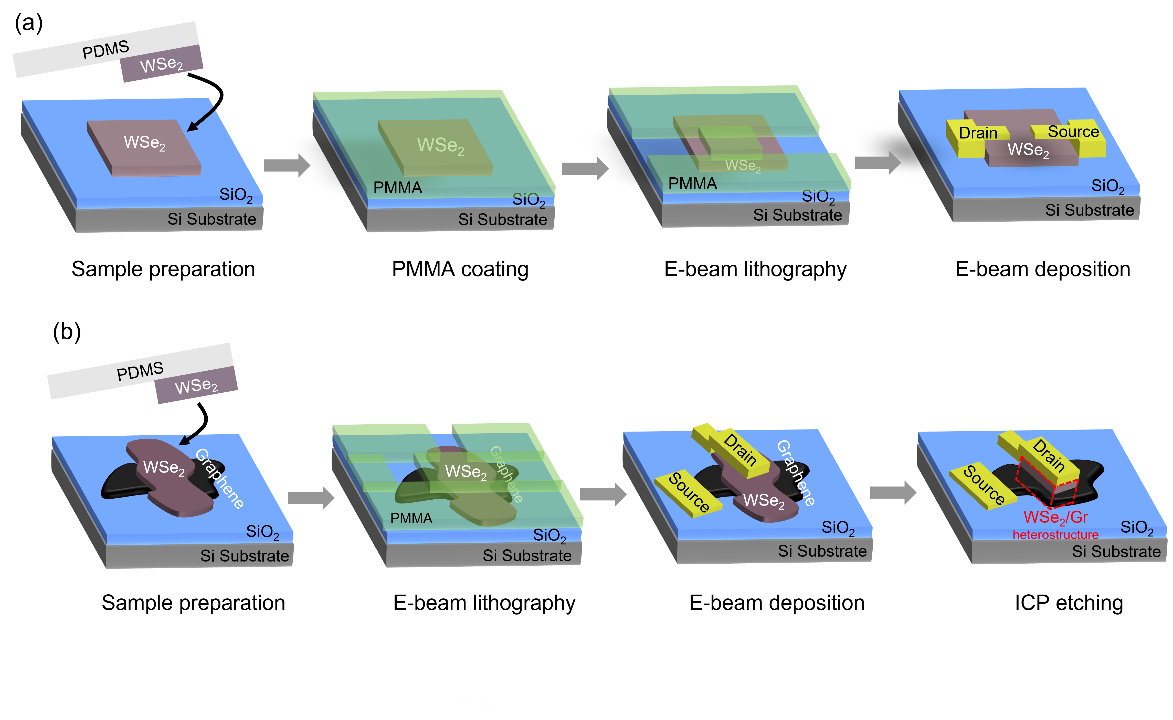


**Figure S1.** Schematic of the process flow for the **(a)** lateral and **(b)** vertical WSe_2_ FET fabrication.

Figure S1 illustrates the lateral and vertical WSe_2_ FETs fabrication process. Herein, both bulk WSe_2_ and bilayer graphene (BLG) were mechanically exfoliated using the Scotch tape method. Subsequently, WSe_2_/BLG heterostructure was formed using the dry transfer method. Inductively coupled plasma etching was performed to eliminate unwanted carrier paths apart from the out-of-plane carrier transport direction. These processes were performed in a glovebox; both O_2_ and H_2_O concentrations were below 0.1 ppm. Next, WSe_2_ alone was transferred onto the SiO_2_/Si substrate for the lateral FET, and the WSe_2_/BLG heterostructure for the vertical FET. Subsequently, source and drain electrodes were formed as follows. Electron resistor layers in polymethyl methacrylate (PMMA) were spin-coated at 2000 rpm for 5 s and 4000 rpm for 35 s. Each layer was baked on a hotplate at 180 °C for 2 min. Au (50 nm) was deposited as the drain and source electrodes using e-beam deposition and patterned using e-beam lithography.

**Supplementary section 2: Bi-layer graphene as a bottom electrode**

**a. Negligible effect of bi-layer graphene layer on carrier concentration**


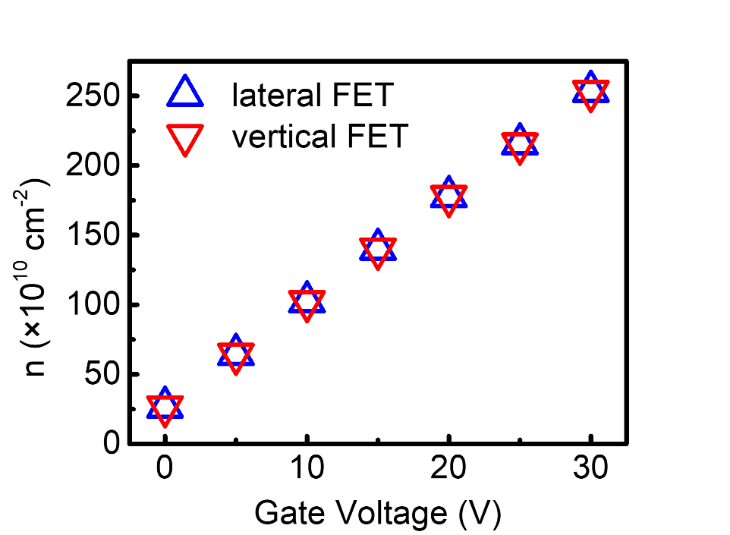


**Figure S2.** Calculated carrier concentration for various applied gate voltages in the lateral (blue triangle) and vertical (red inverted triangle) WSe_2_ FETs.

Figure S2 shows the calculated carrier concentration values for various gate voltages in the lateral and vertical WSe_2_ FETs, determined using the following equation.

$n=\frac{Q}{e}=C_{gate}\times(V_{GS}-V_{th})/e$,

where $C_{gate}$ is the capacitance of the gate dielectric and $e$ is the elementary charge. In the lateral FET, only SiO_2_ was considered and a $C_{gate}$ value of 12.1 nF/cm^−2^ was used. The capacitance of the BLG was considered to be 6.56 μF/cm^−2^. The capacitance value of the BLG is exceptionally large compared with that of SiO_2_, which is negligible. Therefore, $C_{gate}$ presents a very slight difference when considering the SiO_2_ single dielectric and the BLG. Comparing the carrier concentration values of the lateral and vertical WSe_2_ FETs calculated for the same gate voltage, an insignificant difference was observed, thus indicating that the carrier concentration difference due to graphene was negligible.

**b. Gate bias modulation through bi-layer graphene**

Figures S3a and b show OM images of the lateral FETs employing WSe_2_/BLG and WSe_2_ as the channel, respectively. A comparison of the transfer curves of these two devices revealed no significant change, other than an increase in the off current; additionally, gate modulation was observed even in the presence of a bilayer graphene layer.

**
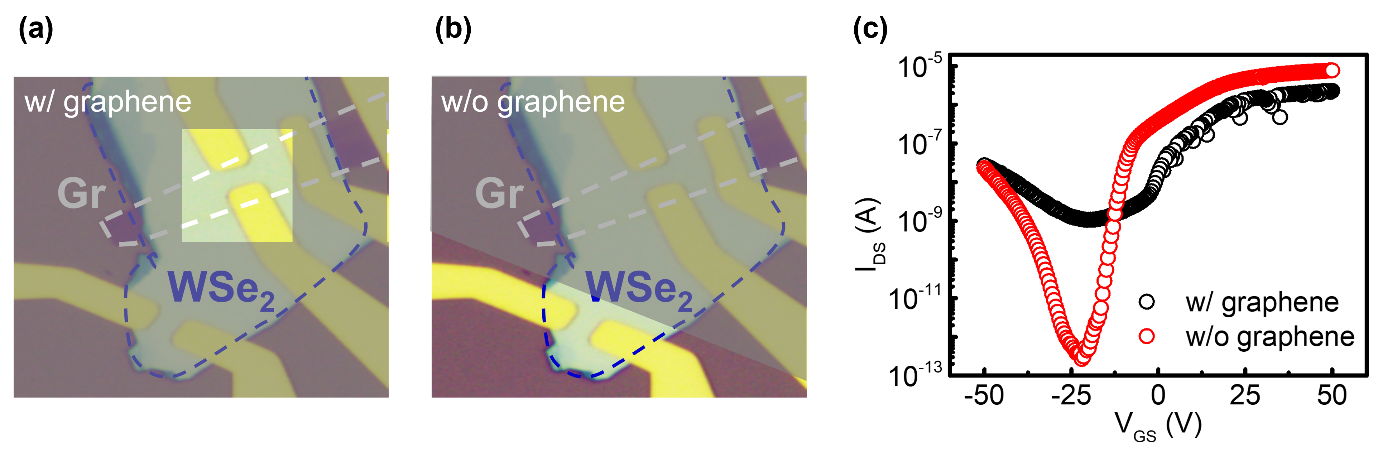
**

**Figure S3.** Optical images of the lateral WSe_2_ FETs **(a)** with a bi-layer graphene layer and **(b)** without graphene. **(c)** Transfer characteristics of these two devices.

**c. Tuning the Schottky barrier at the WSe_2_/Graphene interface through gate voltage**


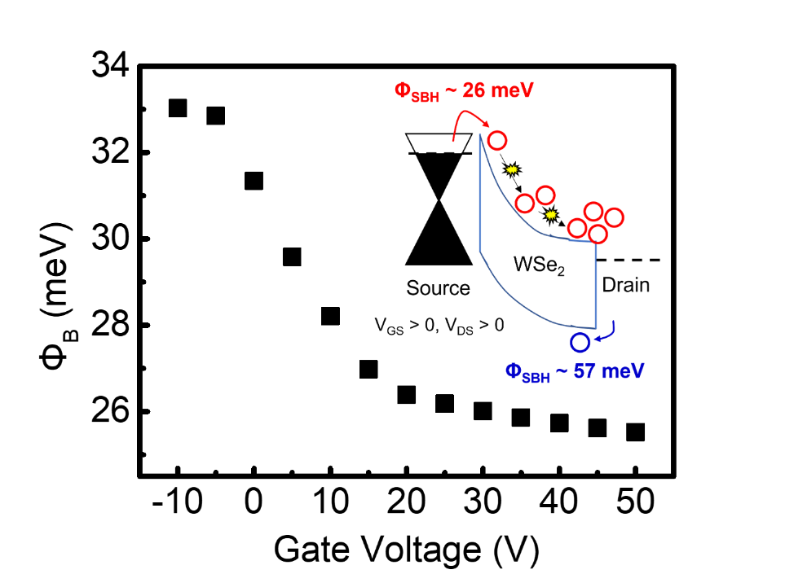


**Figure S4.** Schottky barrier height (Φ_B_) obtained for different values of V_GS_. (Inset: band structure at the WSe_2_/graphene interface for V_GS_ > 0 V)

Figure S4 shows the gate dependence of Φ_B_ at the BLG/WSe_2_ interface, which varied from 28 to 57 meV when the WSe_2_ channel was tuned from the OFF to the ON state. This corresponded to gate modulation of the graphene Fermi level with respect to the WSe_2_ conduction band, as depicted in the inset. The calculation of Φ_B_ was performed based on the transfer characteristics in Fig. 2b, using the following equations.^S1^

|  | $\Phi_{B}=\frac{k_{B}T}{ⅇ}\ln\left( \frac{A^{*}T^{2}}{\bar{J}rⅇv} \right),$ | (S1) |
| --- | --- | --- |
| and | $J=\left( I_{ⅆs}/A_{\dot{j}unC} \right)$. | (S2) |

When V_GS_ = 10 V, the calculated SBH was 28 meV; thus, the electron Schottky barrier height. Considering the hole Schottky barrier height of Au and that the valence band maximum (VBM) of WSe_2_ is 56 meV,^S2^ electrons were confirmed as the majority carriers and contributed to impact ionization in both the lateral WSe_2_ FET and the vertical WSe_2_ FET via the WSe_2_/BLG heterostructure.

**d. Electrical properties of bi-layer graphene**

**
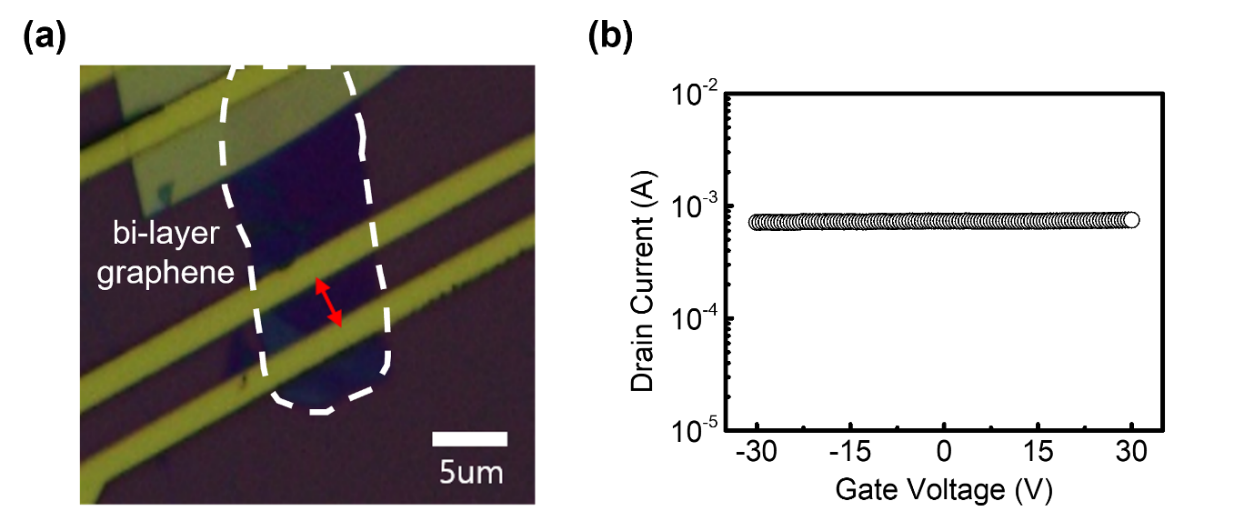
**

**Figure S5**. **(a)** Optical image and **(b)** transfer characteristic of planar bi-layer graphene.

Figure S5 shows an optical image (a) and the transfer characteristics (b) of the planar region of bi-layer graphene, used as the bottom electrode. As shown, it is completely metallic. Consequently, the role of bi-layer graphene as a series resistor is negligible.

**e. Excluding the effect of velocity overshoot**

For vertical transport, as shown in the inset of Fig. S6a, the carriers move semi-ballistically owing to their small channel lengths. Semi-ballistic transport implies that carriers move from one physical end to the other end of a semiconductor within a time smaller than their relaxation time.^S3^ In such a case, carriers manage to obtain steady-state and nonstationary carrier transport, such as velocity overshoot, occurs.^S4^ High applied electric fields can produce a velocity overshoot over small distances by driving electrons to velocities above their corresponding steady-state velocity, as shown in Fig. S6c. Evidently, at low applied fields, the velocity did not significantly overshoot the steady-state value. Whereas as the field increased, the velocity significantly overshot the steady state. The electron transit time in these fields was substantially reduced by the overshoot of electrons at the steady-state velocity.^S5^ Consequently current saturation was not observed without adding the graphene layer, as shown in Fig S6a. However, this velocity overshoot could terminate and decrease when the applied field was further increased. This is because of the transfer of high-energy electrons to satellite valleys, wherein stronger electron–phonon scattering occurs owing to the large density of states. Velocity overshoot can be accomplished by propelling carriers at high energy. High-energy injection at various energies was possible using graphene (inset of Fig. S6b) and different gate voltages. Thus, electrons started with velocities much larger than the steady-state drift velocity. If the electrons are propelled at energies above or near the intervalley threshold, they can be easily accelerated to energies where they will transfer to satellite valleys. This results in a sharp drop in the velocity, with no gain from the overshoot, as shown in Fig. S6d; finally, the current saturates, as shown in Fig. S6b.


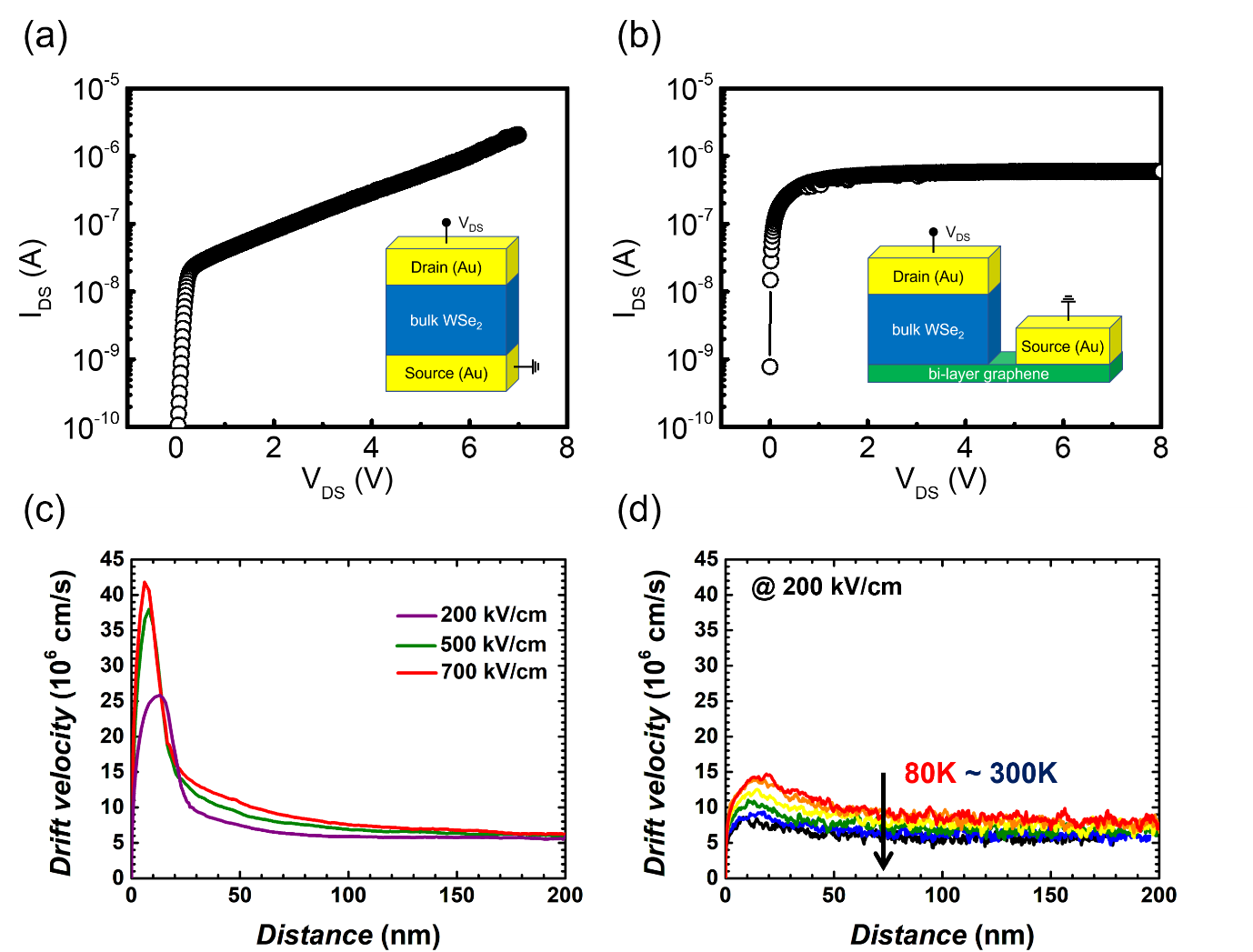


**Figure S6.** Representative I_DS_–V_DS_ characteristics for **(a)** Au/WSe_2_/Au vertical transport and **(b)** Au/WSe_2_/graphene lateral transport. Insets show schematics of each transport. **(c)** Carrier drift velocities as a function of the traveling distance for various applied electric fields, for vertical transport with no graphene. **(d)** Negligible velocity overshoot effect when graphene is inserted as the bottom electrode.

**Supplementary section 3: Demonstration of impact ionization**

**a. Analysis of impact ionization**


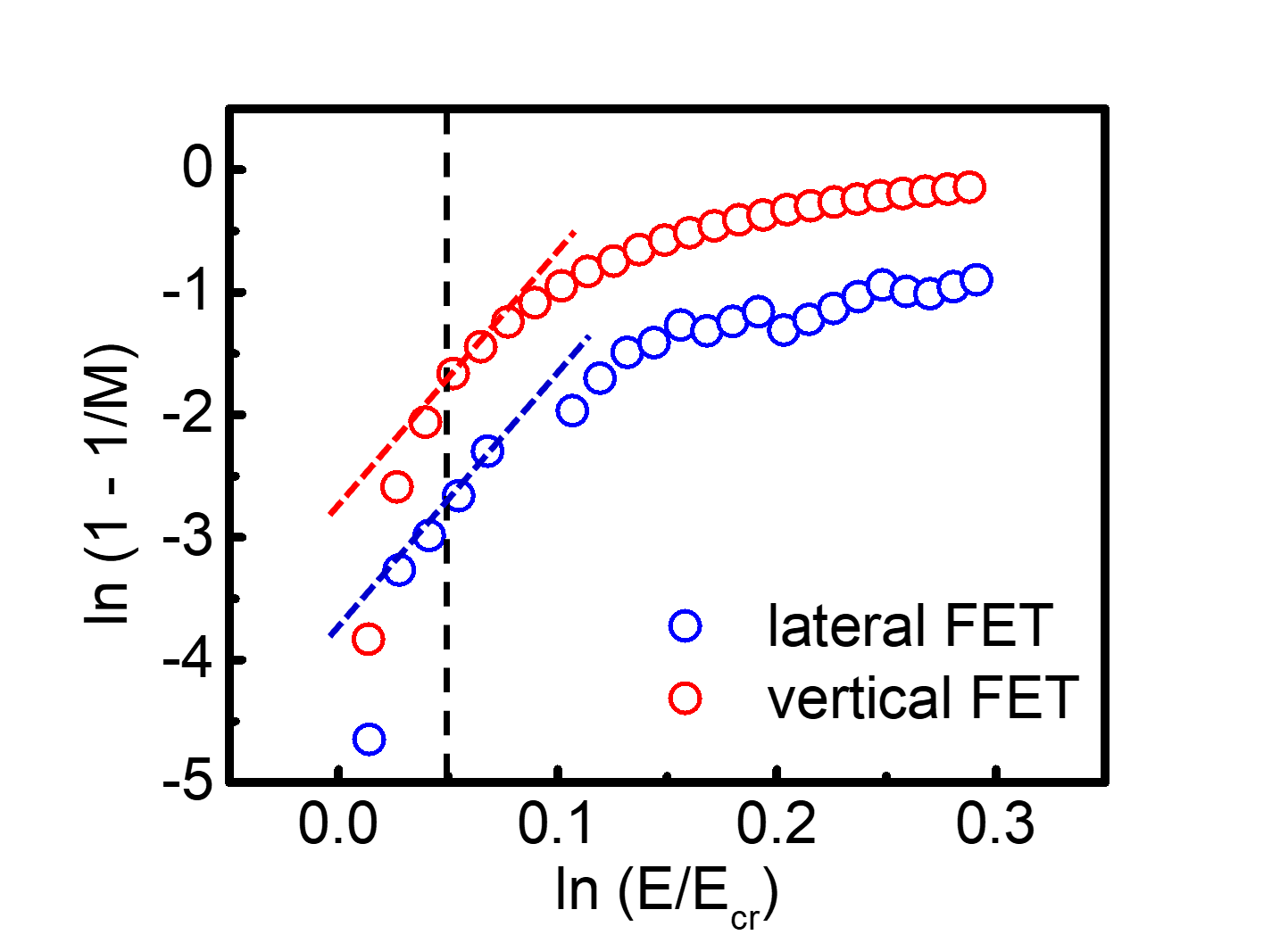


**Figure S7.** 1 – 1/M values versus electric field for the lateral (blue circle) and vertical (red circle) WSe_2_ FETs.

For the impact ionization process, the multiplication factor (M) and electric field (E) satisfy the following equation.^S6^

|  | $M=\left[ 1-\left( \frac{E}{E_{cr}} \right)^{n} \right]^{-1},$ | (S3) |
| --- | --- | --- |

where *n* is an index corresponding to the ionization rate and *E*_CR_ is a critical electric field for the impact ionization process. By fitting the current response with a transformed linear relationship, the following equation is obtained.

|  | $\ln\left( 1- \frac{1}{M} \right)=n \times(\ln\left( E \right)-\ln\left( E_{cr} \right))$. | (S4) |
| --- | --- | --- |

Figure S7 shows the 1 – 1/M values as a function of E/E_cr_ for the lateral and vertical WSe_2_ FETs. As confirmed, the critical electric field was larger in the out-of-plane direction. However, the calculated index *n* (i.e., the slope of the curve at ln(E/E_CR_) = 0.05), related to the ionization rate onset after impact ionization, exhibited a similar value for each carrier transport direction.

**b. Reversible output characteristics during V_DS_ sweeping**

**
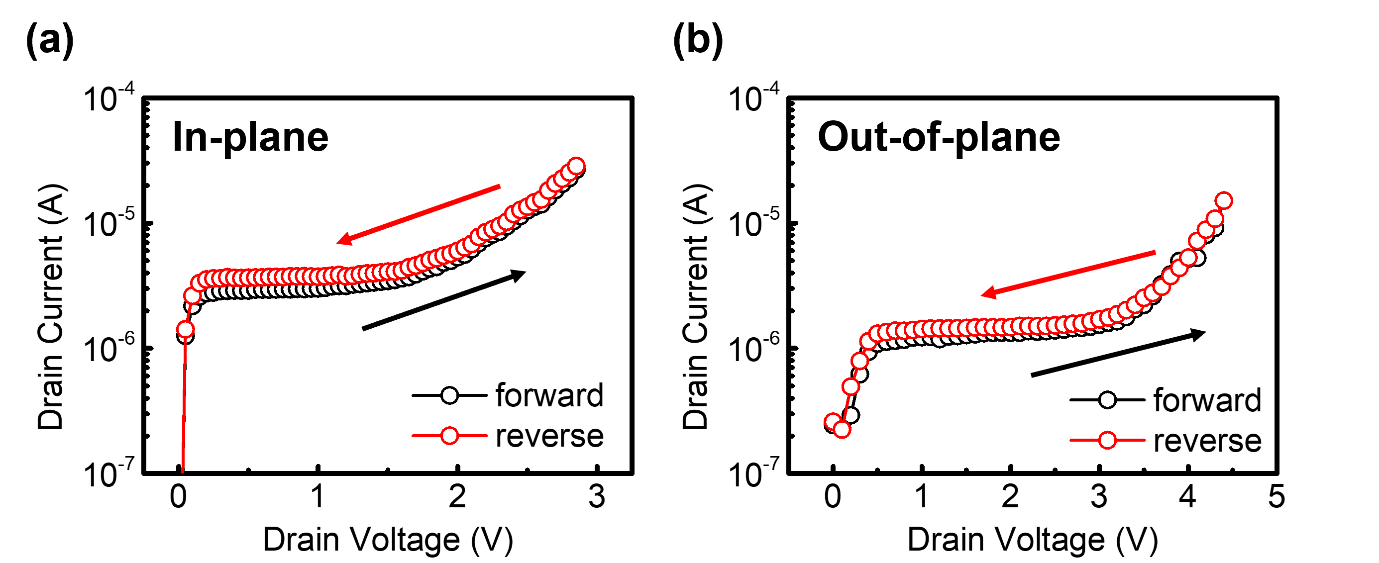
**

**Figure S8.** Hysteresis curves of (a) lateral and (b) vertical WSe_2_ FETs. Sweep directions are marked with arrows.

Figure S8 shows the I_DS_–V_DS_ characteristics of the (a) lateral and (b) vertical WSe_2_ FETs. Reversible curves for each transport direction were measured under multiple V_DS_ sweeps. The small hysteresis indicates that the WSe_2_ channel was not damaged by thermal stress originating from Joule heating after reversible in-plane or out-of-plane impact ionization.

**c. Reliability of the impact ionization process**

The cumulative distributions of the measured critical electric field during repeated impact ionization for in-plane (blue circles) and out-of-plane (red circles) WSe_2_ FETs are shown in Fig. S9. These results were obtained from the cycle-to-cycle data for the same device over several cycles. After tens of cycles, similar critical electric fields were observed for both transport directions. This indicates that no significant degradation occurred, except for slight changes in carrier multiplication.

**
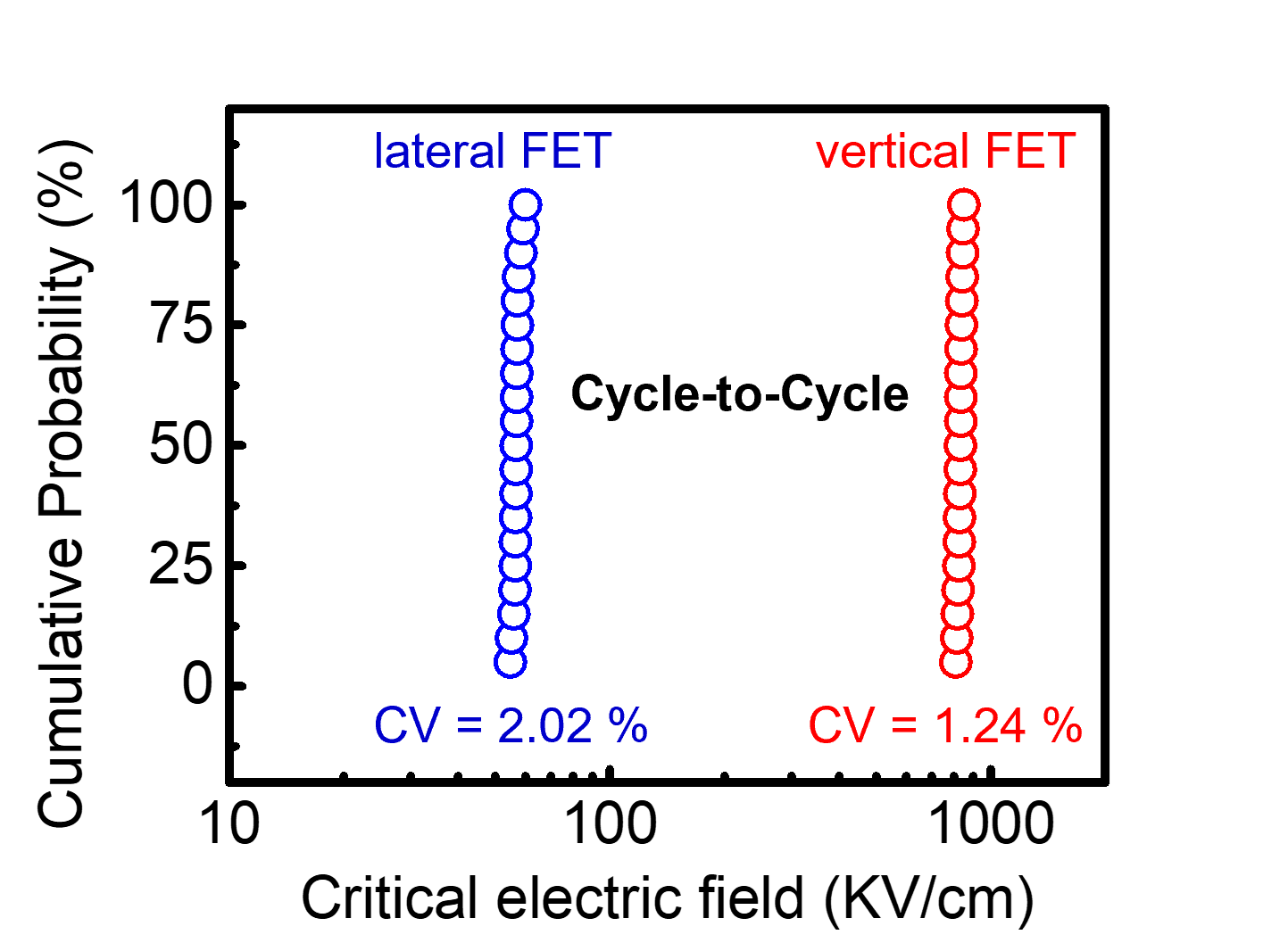
**

**Figure S9.** Critical electric field during repeated impact ionization of the lateral (blue circles) and vertical (red circles) WSe_2_ FETs, shown as cumulative probability curves.

**Supplementary section 4: Monte Carlo simulations**

In this section, SMC and EMC methods were applied to direction-dependent hot-carrier transport in multilayer WSe_2_ FET. Within the statistical simulations, a significant orientation dependence of the ionization rate was observed, which is in good agreement with the interpretation of our experimental results.

**a. Scattering mechanisms in WSe_2_**


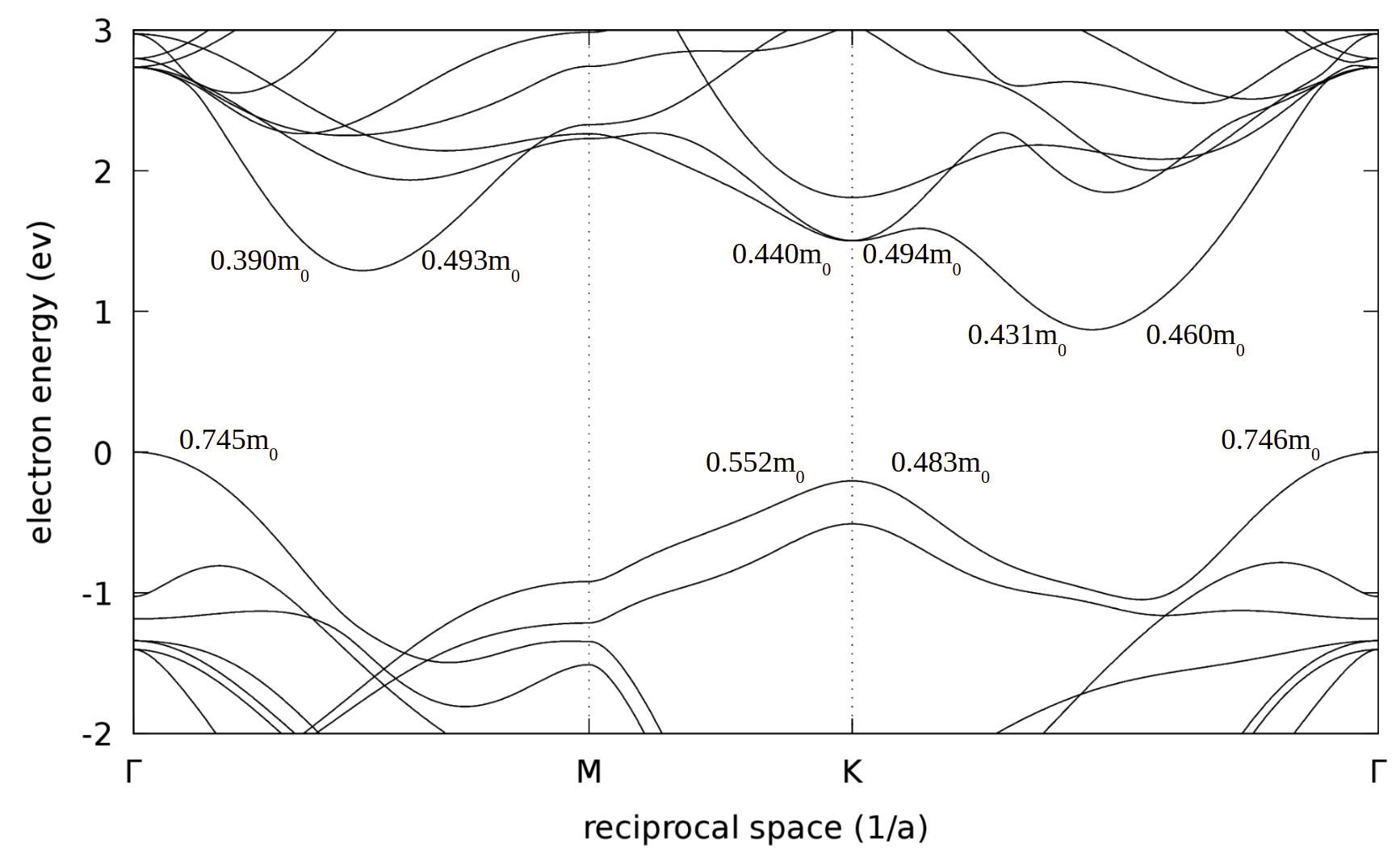


**Figure S10.** DFT-calculated band structure of multilayer WSe_2_ along the Γ-M-K-Γ line in momentum space. The effective mass is shown near each conduction valley in m_0_ units (the mass of the electron); note that the effective hole mass is in fact negative.

Herein, the simplest case was assumed; three conduction band valleys were considered (Q, K, and L in Fig. S10) for the n-type multilayer WSe_2_. Parabolicity in the electronic band was included in the standard form. Table S1 summarizes the band parameters used in our transport simulations, including the valley degeneracy, the separation energy for different conduction valleys, and their corresponding effective masses.

| Conduction valley index | Degeneracy | Effective mass (m0) | Minimum (eV) |
| --- | --- | --- | --- |
| 1(Q) | 6 | 0.445 | 0 |
| 2(K) | 2 | 0.466 | 0.4 |
| 3(L) | 6 | 0.438 | 0.4 |

**Table S1.** Conduction band parameters of multilayer WSe_2_

The Monte Carlo procedure generates random numbers to represent the time at which the electrons drift freely in the electric field.^S7–10^ Combined with deterministic processes, the trajectories of carriers were simulated until they reached the threshold energy for impact ionization. This was performed with a comprehensive knowledge of the scattering mechanisms in the system. For hot-carrier transport in multilayer WSe_2_, phonon scatterings were set up to determine the trajectories of the electrons in three-dimensional space. Acoustic phonon, polar optical phonon, and nonpolar optical phonon scatterings were considered in each conduction valley.

Based on Fermi’s golden rule,^S11^ the deterministic scattering rate from a state **k** to a state **k’** is given by:

|  | $W_{\text{k}\text{k}^{\boldsymbol{'}}}=\frac{2\pi}{\hbar}\left\vert C\left( \mathbf{q} \right) \right\vert^{2}\Delta(\epsilon(\mathbf{k}),\epsilon(\mathbf{k}^{\mathbf{'}}))$, | (S5) |
| --- | --- | --- |

where $q=\left| \mathbf{q} \right|=\left| \mathbf{k}-\mathbf{k}' \right|$ and the factor $\Delta(\epsilon(\mathbf{k}),\epsilon(\mathbf{k}^{\mathbf{'}}))$ is given by:

|  | $\Delta(\epsilon,\epsilon^{'})=N_{q}\delta(\epsilon-\epsilon^{'}+\hbar\omega_{q})+(N_{q}+1)\delta(\epsilon-\epsilon^{'}-\hbar\omega_{q})\Theta(\epsilon-\hbar\omega_{q})$, | (S6) |
| --- | --- | --- |

where $\hbar_{q}$ is the phonon energy with an occupation number $N_{q}=1/\left[ \exp\left( \beta\omega_{q} \right)-1 \right]$. The first (second) term in Eq. (S6) corresponds to the absorption (emission) of a phonon. The matrix element $C\left( \mathbf{q} \right)$ was determined by different phonon scattering mechanisms.

For acoustic phonon scattering via deformation coupling:

|  | $\left\vert C(\mathbf{q}) \right\vert^{2}=\frac{D^{2}\hbar q}{2u_{l}\rho}$ , | (S7) |
| --- | --- | --- |

where *D* is the deformation-potential coupling constant; $u_{l}$ is the longitudinal sound velocity; and *ρ* is the mass density. For a polar LO phonon:

|  | $\left\vert C(\mathbf{q}) \right\vert^{2}=\frac{2\pi e^{2}\hbar\omega_{0}}{q^{2}}\left( \frac{1}{{}_{\infty}}-\frac{1}{{}_{0}} \right)$, | (S8) |
| --- | --- | --- |

where ℏω_0_ equals 32 meV (E_g_ mode) for the LO-phonon energy; κ_∞_ = 7.7 is the optical dielectric constant; κ_0_ = 7.8 is the static dielectric constant; and *e* is the electron charge. For a zero-order nonpolar optical phonon:

|  | $\left\vert C(\mathbf{q}) \right\vert^{2}=\frac{D_{0}^{2}\hbar}{2\rho\omega_{0}}$ , | (S9) |
| --- | --- | --- |

where *D*_0_ is the deformation-potential coupling constant and ω_0_ is the frequency of the nonpolar optical phonon.

For the lateral device, only the lowest conduction valley was considered in the 2D matrix element *C*(**q**). For acoustic phonon scattering via deformation coupling:

|  | $\left\vert C(\mathbf{q}) \right\vert^{2}=\frac{D^{2}\hbar q}{2\rho u_{l}A}$ , | (S10) |
| --- | --- | --- |

where *D* is the deformation-potential coupling constant; *A* is the area; and *ρ* and $u_{l}$ are the mass density and longitudinal sound velocity, respectively. For a polar LO phonon:

|  | $\left\vert C(\mathbf{q}) \right\vert^{2}=\frac{2\pi e^{2}\omega_{0}}{qA}\left( \frac{1}{{}_{\infty}}-\frac{1}{{}_{0}} \right)$, | (S11) |
| --- | --- | --- |

where ℏω_0_ equals 32 meV (E_g_ mode) for the LO-phonon energy; κ_∞_ = 15.6 is the optical dielectric constant; κ_0_ = 15.9 is the static dielectric constant; and *e* is the electron charge. For a zero-order nonpolar optical phonon:

|  | $\left\vert C(\mathbf{q}) \right\vert^{2}=\frac{D_{0}^{2}\hbar}{2\rho\omega_{0}A}$ , | (S12) |
| --- | --- | --- |

where *D*_0_ is the deformation-potential coupling constant and ω_0_ is the frequency of the nonpolar optical phonon.

**b. Monte Carlo simulation result for out-of-plane impact ionization**

As the applied field was further increased, avalanche multiplication occurred because of impact ionization. The threshold energy (E_I_) was estimated to be approximately 1.2 eV for multilayer WSe_2_, corresponding to a field strength of 140 kV/cm, as compared with the SMC simulated electron energy result shown in Fig. 3d. In particular, Fig. S11a shows the EMC simulated electron average energy in WSe_2_ as a function of distance for large applied fields, wherein electrons were injected cold (zero initial energy) at the cathode; unlike the small electric field cases shown in Fig. S11b, the electrons could not obtain a steady state within the 200 nm channel length for field strengths larger than 200 kV/cm, even under the propelling energy condition.


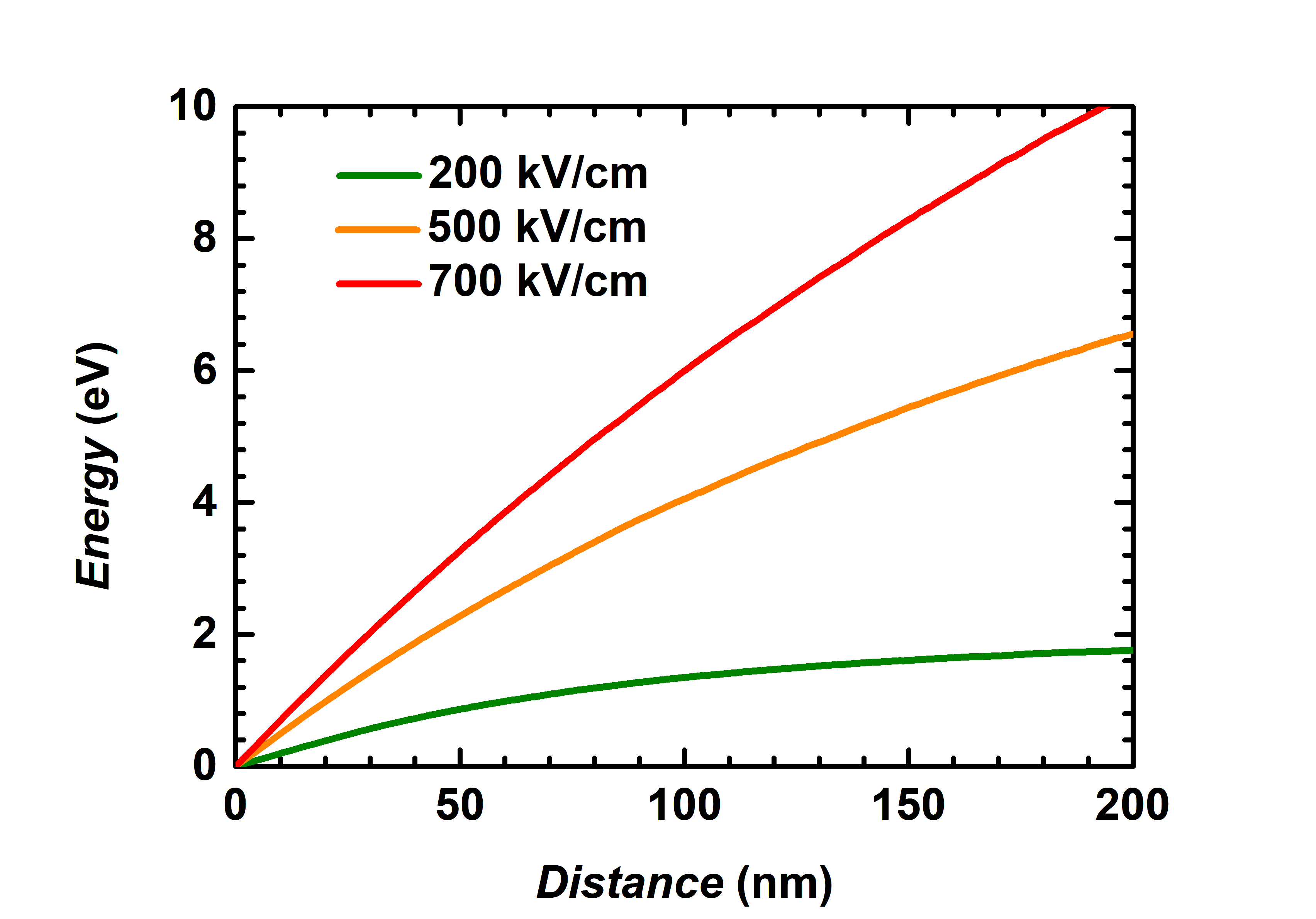

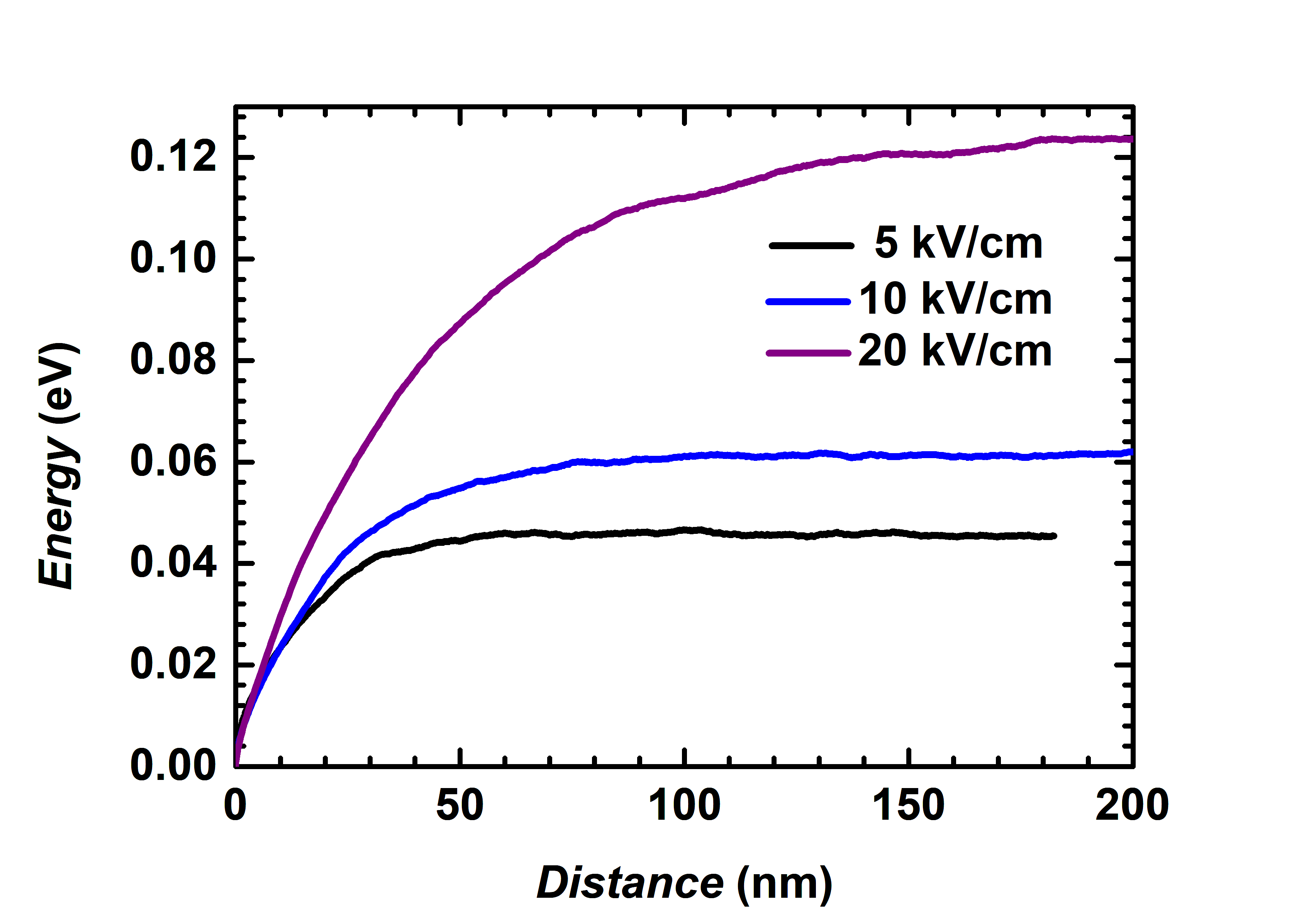


**Figure S11.** Average electron energy versus device distance in multilayer WSe_2_ for out-of-plane transport under (a) large and (b) small electric fields at a temperature of 300 K.

Figures S12 and S13 show the SMC-simulated average energy and drift velocity as a function of the applied field in WSe_2_ for various temperatures. Both quantities increased with decreasing operating temperature owing to suppressed phonon scattering. In particular, to obtain the same average energy, a lower applied field was required when the temperature decreased; this trend became more significant for a larger average energy. This indicates that for the experimentally measured temperature-dependent critical electric field, temperature plays a more important role at larger threshold energies (E_I_).


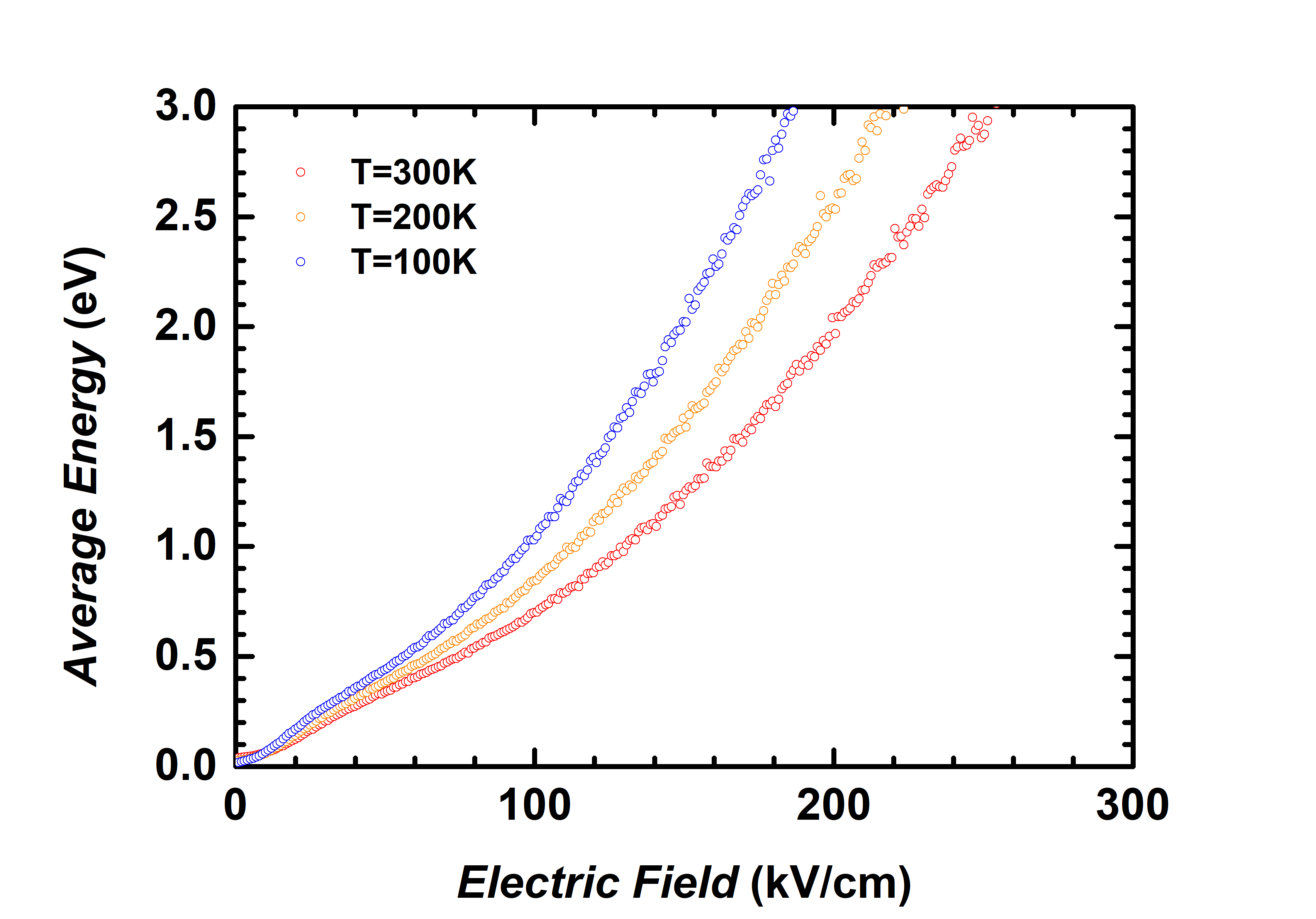


**Figure S12.** Average electron energy versus the applied field in multilayer WSe_2_ at different temperatures.


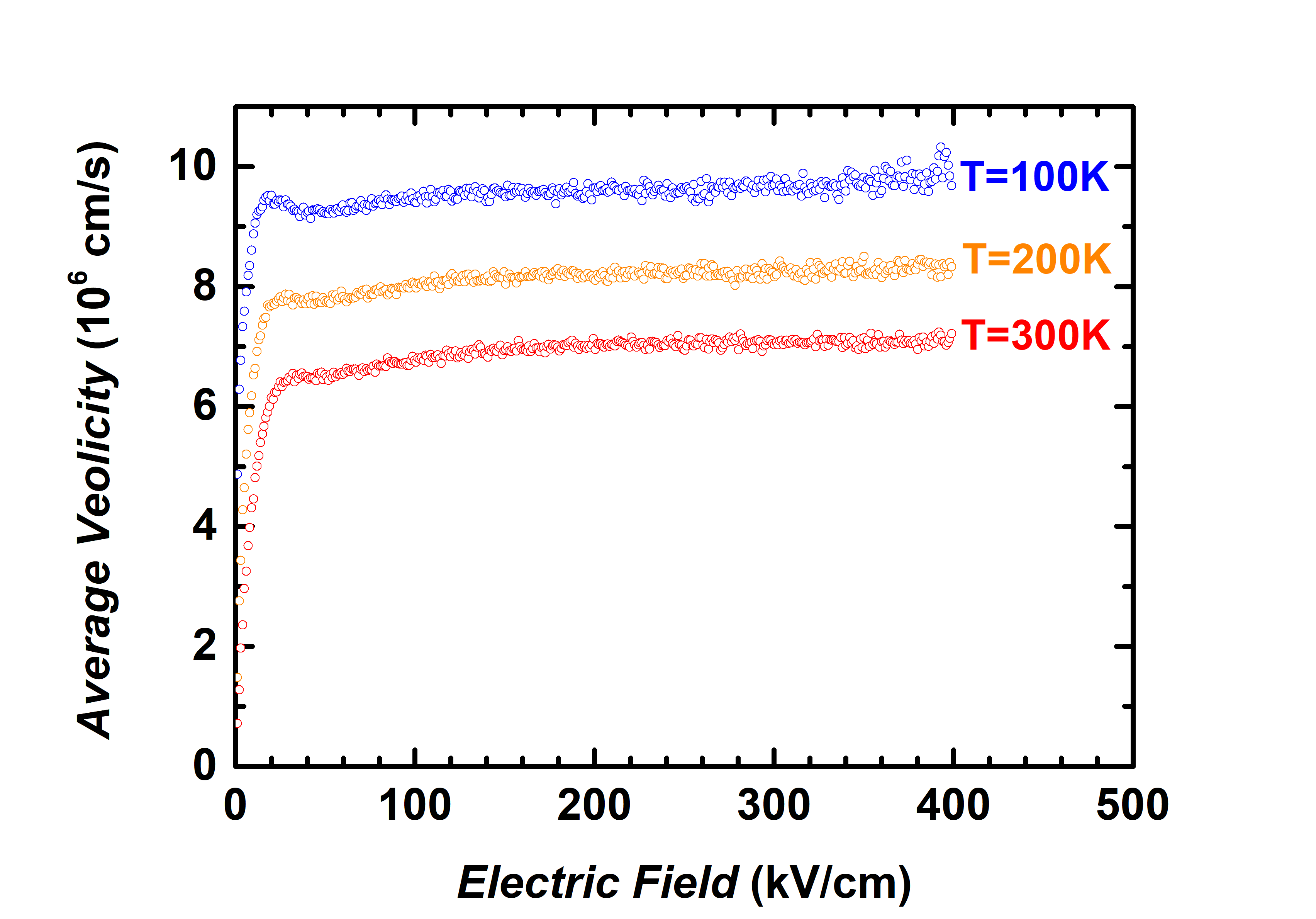


**Figure S13.** Average drift velocity versus the applied field in multilayer WSe_2_ at different temperatures.

**c. Monte Carlo simulation result for in-plane impact ionization**

For lateral devices fabricated using multilayer WSe_2_, the in-plane carrier transport guarantees steady-state behavior of the motion of a carrier in the 2D inversion layer. Thus, its transport was monitored using the single electron Monte Carlo (SMC) method. The resulting scattering rates of the different phonons are plotted in Fig. 3a as a function of the carrier energy at 300 K. The different curves in Fig. 3a show the contributions of acoustic phonons, polar LO phonons, and nonpolar optical phonons to the total scattering rate. The total scattering rate was dominated by nonpolar optical phonon scattering. The sharp cusp observed in the curve at an optical phonon energy of 32 meV was because the carriers possessed sufficient energies to emit optical phonons. Unlike the 3D scattering rates of the vertical device shown in Fig. 3c, the total scattering strength in Fig. 3a weakened as the electron energy increased above 32 meV (the optical phonon energy). An energy runaway occurred, which is thought to be the cause of the results shown in Fig. 3b, where the breakdown electric field (approximately 50 kV/cm at 300 K) was much smaller than that in the vertical device (approximately 700 kV/cm at 300 K).

**Supplementary References**

[S1] Nazir, G.; Khan, M. F.; Aftab, S.; Afzal, A. M.; Dastgeer, G.; Rehman, M. A.; Seo, Y.; Eom, J., Gate tunable transport in Graphene/MoS_2_/(Cr/Au) vertical field-effect transistors. *J. Nanomater.* **2017,** *8* (1), 14.

[S2] Jung, D. H.; Kim, T., Quantification of Schottky barrier height and contact resistance of a Au electrode on multilayer WSe_2_. *J. Korean. Phys. Soc.* **2022,** *80* (4), 307-310.

[S3] Teitel, S. L.; Wilkins, J., Ballistic transport and velocity overshoot in semiconductors: Part I—Uniform field effects. *IEEE Trans Electron Devices* **1983,** *30* (2), 150-153.

[S4] Jyegal, J., Velocity overshoot decay mechanisms in compound semiconductor field-effect transistors with a submicron characteristic length. *AIP Adv.* **2015,** *5* (6), 067118.

[S5] Wraback, M.; Shen, H.; Rudin, S.; Bellotti, E., Experimental and theoretical studies of transient electron velocity overshoot in GaN. *Phys. Status Solidi B.* **2002,** *234* (3), 810-816.

[S6] Lei, S.; Wen, F.; Ge, L.; Najmaei, S.; George, A.; Gong, Y.; Gao, W.; Jin, Z.; Li, B.; Lou, J., An atomically layered InSe avalanche photodetector. *Nano Lett.* **2015,** *15* (5), 3048-3055.

[S7] Biagi, S., Monte Carlo simulation of electron drift and diffusion in counting gases under the influence of electric and magnetic fields. *Nucl. Instrum. Methods. Phys. Res. B NUCL INSTRUM METH A.* **1999,** *421* (1-2), 234-240.

[S8] Urteaga, I.; Bugallo, M. F.; Djurić, P. M. In Sequential Monte Carlo methods under model uncertainty, *IEEE Statistical Signal Processing Workshop (SSP*), 2016, 1-5.

[S9] Ando, Y.; Cappy, A., Ensemble Monte Carlo simulation for electron transport in quantum wire structures. *J. Appl. Phys.* **1993,** *74* (6), 3983-3992.

[S10] Moko, M.; Moková, A., Ensemble Monte Carlo simulation of electron-electron scattering: Improvements of conventional methods. *Phys. Rev. B.* **1991,** *44* (19), 10794.

[S11] Zhang, J.-M.; Liu, Y., Fermi’s golden rule: its derivation and breakdown by an ideal model. *Eur. J. Phys.* **2016,** *37* (6), 065406.
